# Unraveling *IFI44L*’s biofunction in human disease

**DOI:** 10.3389/fonc.2024.1436576

**Published:** 2024-12-16

**Authors:** Juan Du, Hui Luo, Shuang Ye, Hui Zhang, Zhen Zheng, Kaitai Liu

**Affiliations:** The Affiliated Lihuili Hospital of Ningbo University, Ningbo, China

**Keywords:** biomarker, diagnosis, immune response, interferon-induced protein 44-like (IFI44L), therapeutic target, tumor

## Abstract

Interferon-induced protein 44-like (*IFI44L*) is regarded as an immune-related gene and is a member of interferon-stimulated genes (ISGs). They participate in network transduction, and its own epigenetic modifications, apoptosis, cell-matrix formation, and many other pathways in tumors, autoimmune diseases, and viral infections. The current review provides a comprehensive overview of the onset and biological mechanisms of *IFI44L* and its potential clinical applications in malignant tumors and non-neoplastic diseases.

## 1 Introduction

Interferon-induced protein 44-like (*IFI44L*) is an identified gene that is involved in various cancerous and non-cancerous diseases including oral cancer (OC), renal cell carcinoma (RCC), Sjogren’s syndrome (SS), and systemic lupus erythematosus (SLE) (1–4). It participates in numerous biological events, such as innate immune response, inflammation, cell death, phosphorylation, tumor cell proliferation, and cell signaling. It plays a crucial role in defending organisms against bacteria, viruses, and tumors (5–7). It is frequently used as a biomarker for identifying and diagnosing diseases and also serves as a possible target for treating many diseases (8, 9). For instance, different levels of DNA methylation in *IFI44L* can distinguish between SLE patients and healthy individuals (8), as well as between patients with viral infections and those with bacterial infections (9). Therefore, *IFI44L* shows a growing significance as a potential biomarker for the rapid and non-invasive detection of diseases.

The prognosis for tumors remains poor despite the implementation of many innovative therapeutic approaches; they continue to present a significant global health risk (10). In cancer research, the need for biomarkers to aid in identifying and treating diseases has become increasingly significant. The implementation of precise therapeutic targets can substantially enhance patients’ survival rates and significantly improve the rate at which diseases are detected in clinical practice (11). A recently identified gene, *IFI44L* is present in various types of tumors, including pancreatic ductal adenocarcinoma (PDAC), laryngeal cancer, hepatocellular carcinoma (HCC), and lung cancer (LC). Its expression levels have shown significant differences in these tumor types (6, 12–14). In lung cancer cells, it can activate the JAK/STAT1 signaling pathway to promote apoptosis (14), and upregulation in head and neck squamous cell carcinoma cells can promote cancer cell proliferation and invasion (15). Among non-tumor diseases, *IFI44L* was found to be strongly associated with immune disorders, especially SLE, SS, rheumatoid arthritis (RA) and various viral infectious diseases (8, 16–19). This gene contributes to immune functions in different immune cells, including T cells, monocytes, and B cells. It inhibits tumor growth, prevents viral and bacterial replication, and reduces inflammation progression via its involvement in signal transduction, and genetic, and DNA methylation level of *IFI44L* (5, 16, 20, 21).

Thus, this review provides a detailed overview of *IFI44L*, covering its biological functions and mechanisms, as well as its correlation with tumor and non-tumor diseases. It highlights the potential of *IFI44L* as a biomarker in the diagnosis and treatment of human diseases.

## 2 Biogenesis and mechanism of *IFI44L*

*IFI44L* is one of the ISGs and belongs to the *IFI44* family (22). This protein is situated on chromosome 1p31.1 and consists of 452 amino acids (23). Its molecular weight is approximately 47 kDa. Based on a study on Crassostrea gigas, *IFI44L* (also known as Cg*IFI44L*-1) is the most abundant protein in blood cells, primarily localized in the cytoplasm, and its cDNA has an Open Reading Frame length of 1437 base pairs. It encodes a 479 amino acid peptide with the TLDc and MMR_HSR1 domains and it has the highest content in blood cells, mainly distributed in the cytoplasm (24).

It always participates in congenital immune responses (25), and has a strong anti-virus specificity (26). It is also essential for antiviral, antibacterial, and antitumor activity, in other biological processes, and is associated with several diseases (14, 27). Its involvement in innate immunity, inflammation, histone modification, methylation, programmed cell death, phosphorylation, cell proliferation, extracellular matrix (ECM) formation, and cell signaling has been supported by accumulating evidence (5, 28). Here, *IFI44L*’s biological functions and mechanisms, including genetic epigenetic modification, specific gene regulation and signal transduction, and cell-matrix formation, are summarized in this review.

### 2.1 Participate in specific gene regulation and signal transduction

**Figure 1:**
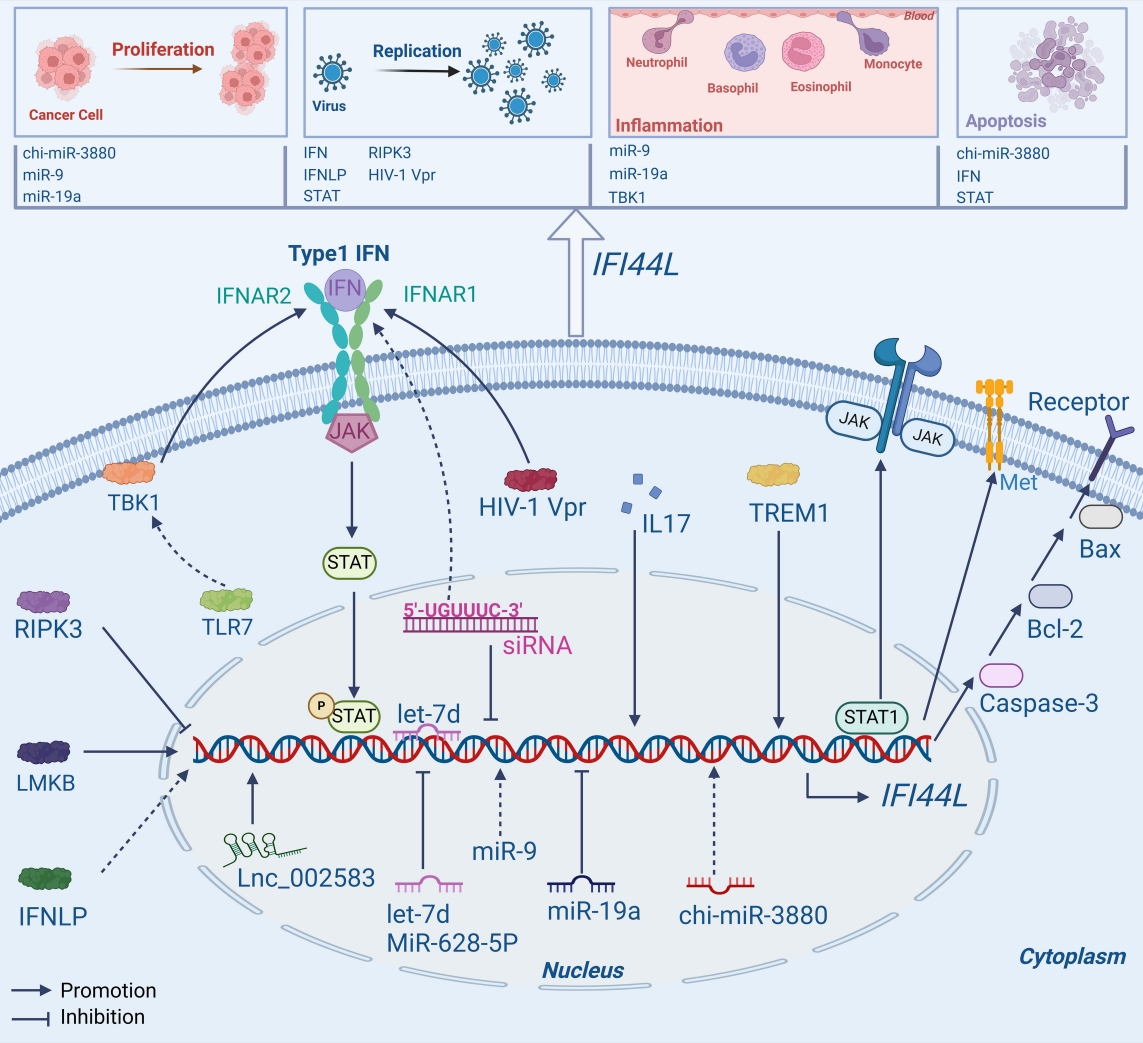
Mechanisms of *IFI44L*. *IFI44L* is not only a target gene for upstream molecules like RNA and protein, but can also affect some pathways such as JAK/STAT1, Type1 IFN, and Caspase-3/Bcl-2/Bax signaling pathway, playing a vital role in human diseases.

Commonly, *IFI44L* exerts its important biological functions as a downstream target gene and signal transduction molecule as shown in **Figure 1**.

#### 2.1.1 As a downstream target gene

Various studies have shown that *IFI44L* has the potential to function as a downstream target gene, binding with some specific proteins or genes. For example, in a recent study, researchers used RNA sequencing to investigate the underlying mechanisms. The findings revealed a connection between Lnc_002583, a type of Long non-coding RNAs, and the expression of *IFI44L*. It was observed that Lnc_002583 may have a positive impact on *IFI44L* expression, suggesting a potential synergistic regulatory function in the growth and development of *Leqiong cattle* (29).

In plasmacytoid dendritic cells (pDCs), *IFI44L* expression was reduced following BX795 (TBK1 inhibitor) therapy. TANK-binding kinase (TBK1) can produce IFN-I and then promote the production of ISGs including *IFI44L*, thereby contributing to the suppression of viral replication (30). It has also been observed that human B and T lymphocyte cells express Limkain B (LMKB). A decrease in LMKB expression may stimulate the expression of *IFI44L*, *IFI44L* and LMKB can interact to control mRNA stability and reduce the inflammatory response, indicating that LMKB and *IFI44L* may interact to have an anti-inflammatory effect on B cells and T cells (31). In a recent study, Ji, X., et al. used human umbilical vein endothelial cells (HUVECs) to investigate the effects of a mimic control and let-7d, a specific type of microRNAs. Interestingly, the findings revealed that the transfection of let-7d led to a decrease in the expression of *IFI44L*. Further validations revealed that let-7d could directly bind to the 3’ untranslated region (3′-UTR) of *IFI44L* and cause a negative regulatory effect on its expression. This inhibition of *IFI44L* activity resulted in the suppression of endothelial cell propagation and migration, which in turn affects the development of inflammation and consequently influences the progression of diseases associated with vascular inflammation (32).

The expression of *IFI44L* was substantially downregulated in A549, HCC827, and CNE2 cells in the miR-19a (a type of microRNA) overexpression group. However, the inhibition of miR-19a facilitates the immuno-oncological activity of *IFI44L* within cellular compartments. The differential expression patterns of miR-19a and *IFI44L* may be harnessed as biomarkers for the prognostication and diagnostic evaluation of malignancies, thereby providing insights into the trajectory of disease progression and the efficacy of therapeutic interventions (33). In CNE2 cells, miR-9 was overexpressed to investigate its downstream target gene, and *IFI44L* emerged as the most significantly upregulated gene. Following the application of miR-9 inhibitors, *IFI44L* expression was downregulated, indicating that miR-9 can possibley target and regulate *IFI44L*, thus affecting the relationship between nasopharyngeal carcinoma (NPC) and inflammation (34). To promote the propagation, migration, and invasion of osteosarcoma (OS) cells, miR-9 can bind to the 3′-UTR of *IFI44L*, thereby targeting and inhibiting its expression (35). Recent studies have indicated that in head and neck squamous cell carcinoma (HNSCC) cells, the knockdown of *IRF1* can reduce the expression levels of *IFI44L* and suppress cell proliferation and invasiveness. The overexpression of *ACSL4* disrupts interferon signaling, enhances the expression of *IFI44L*, and promotes the proliferation and invasiveness of HNSCC cells. However, the specific mechanisms of their roles in tumor development require further investigation (15).

As a target gene, *IFI44L* is differentially expressed in a substantial number of virus-infected cells and, in certain circumstances, displays antiviral activity (36–39). Studies indicated that high levels of *IFI44L* have been observed to impede the replication and dissemination of the Japanese encephalitis virus (JEV), the expression of *IFI44L* can be inhibited by interacting with serine/threonine-protein kinase 3 (RIPK3) receptors. Therefore, inhibiting the expression of *IFI44L* would lead to the propagation of JEV in neurons (40). The expression of *IFI44L* was upregulated in IFN-like protein (IFNLP) treated haemocytes, but significantly downregulated after the deletion of *IFI44L* and *STAT*, suggesting that IFNLP and *STAT* participate in the antiviral immune response of oysters by targeting *IFI44L* (24). Therefore, inhibiting viral replication may be possible through the functions of *IFI44L*.

In addition to being influenced by upstream molecules, this gene can also be affected by LTR-retrotransposons. The 011052702-MALR1044uL locus has been identified in the 3′-UTR of *IFI44L* and has the potential to impact the expression of *IFI44L* in specific circumstances (41). However, these studies only identified the molecules that interact with *IFI44L*, they laid the groundwork for a deeper mechanistic study of this gene.

#### 2.1.2 As a signal transduction molecule

This *IFI44L* gene can interact with proteins and also functions as a signaling molecule, contributing to various signaling pathways and influencing the progression of diseases. It is a significant molecular biomarker that has a crucial role in regulating the Interferon (IFN) signaling pathway. Its impact extends to influencing viral replication and bacterial growth (42).

Studies have shown that in dendritic cells (DCs) infected with human immunodeficiency virus type 1 (HIV-1), a protein called Vpr can regulate *IFI44L*. This pleiotropic protein can stimulate *IFI44L*, leading to the production and activation of the type I IFN signaling pathway in human DCs (25). This offers new perspectives on the therapeutic management of HIV-1 infection. Wallen, A.J., et al. discovered that specific short interfering RNA (siRNA) contains the hexamer region 5′-UGUUUC-3′; mutation of this region inhibits the expression of *IFI44L*, which is essential for the IFN signaling pathway (43). During an *in vitro* infection system with hepatitis B virus (HBV), IFN-γ and IFN-α can remarkably regulate *IFI44L* expression. It has the potential to resist the HBV virus by suppressing certain pathways involved in nuclear factor-κβand signal transducers and transcription 1 (44). In a study focusing on *Leishmania raziliensis*, the researchers classified the patients into two groups (high and low expressions) based on their levels of IFN-γ secretion. The findings indicated that *IFI44L* displayed significant expression in the group characterized by elevated levels of IFN-γ, and showed a correlation with the signaling pathways of IL17 and TREM1 (45).In a recent study conducted by Wahadat, M.J. et al., it was revealed that the *IFI44L* expression increased with TLR7, RNA, and DNA binding receptors in CD14+ monocytes of patients with Childhood-onset SLE (cSLE). These receptors were found to have a role in promoting *IFI44L* sensitization via the TBK1 signaling pathway (46).

Many interferon genes may be modulated by chi-miR-3880, which is classified as a microRNA, in goat mammary epithelial cells (GMECs). Furthermore, chi-miR-3880 and IFNγ (including *IFI44L*) can modulate cell apoptosis *via* the Caspase-3/Bcl-2/Bax pathways, in addition to regulating lipid forfmation and cell growth via the PI3K/AKT/mTOR pathway (47). In human laryngeal carcinoma HEp2 cells, the expression of *IFI44L* is upregulated and can ultimately suppress the progression of laryngeal carcinoma by triggering apoptosis of laryngeal carcinoma cells through interaction with IFN-α (6). Nevertheless, the specific mode of interaction between them has not been studied. In LC cells, *IFI44L* can directly bind to STAT1 (a type of protein), promote STAT1 phosphorylation and activate JAK/STAT1 signaling pathway, causing cell apoptosis, the overexpression of *IFI44L* exerted a significant inhibitory effect on cellular proliferation and concomitantly induced apoptosis. Conversely, the depletion of *IFI44L* resulted in an enhancement of cellular proliferative capacity and a correspondingly substantial decrease in apoptotic cell death (14). The ability of *IFI44L* to stimulate cellular apoptosis indicates its considerable promise as a therapeutic agent for cancer.

Substantially, *IFI44L*, as a downstream target gene, has the potential to play a key role in different signaling cascades. Some specific proteins and non-coding RNAs, such as TBK1, LMKB, Lnc_002583, and miR-19a, may interact with *IFI44L* or influence its expression (29–31, 33). As a signal transduction molecule, it can regulate signaling pathways including IFN signaling, Caspase-3/Bcl-2/Bax apoptosis-related pathways, and JAK/STAT1 signaling (14, 44, 47). Unfortunately, the specific chemical mechanism has not been adequately investigated, posing a new avenue for future research.

**Figure 2:**
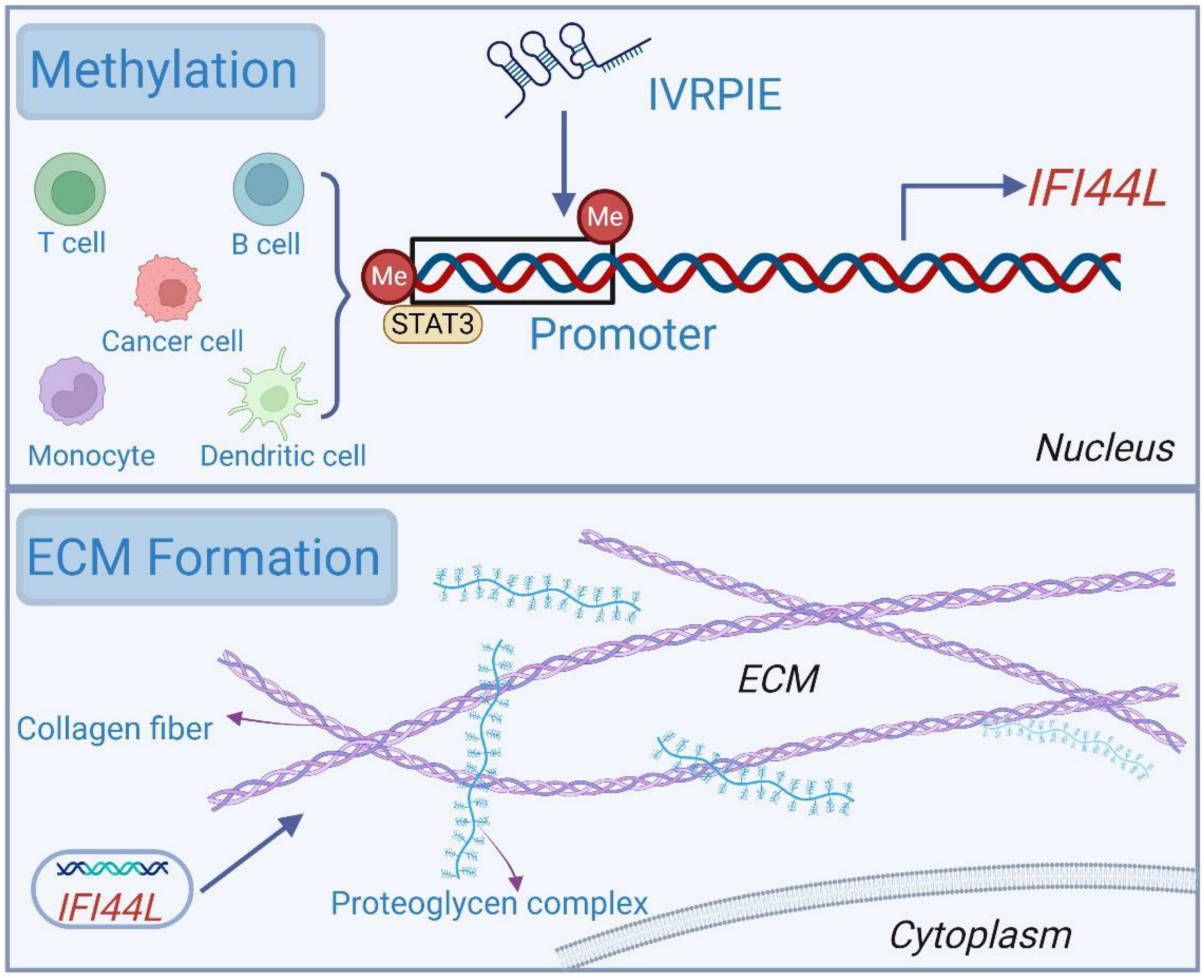
Other mechanisms of action of *IFI44L. IFI44L* mainly participates in biological activity by altering expression through methylation of its own promoter region, and can participate in the formation of ECM.

Besides, *IFI44L* can also alter its own expression through methylation in its promoter region, thereby affecting biological processes such as matrix formation as displayed **Figure 2**.

### 2.2 Methylation of *IFI44L*

The *IFI44L* gene has multiple biological functions, serving as both a target gene and a signal transduction molecule. It has a significant role in immune diseases, particularly concerning methylation and histone modification. These processes are frequently linked to the development of SLE (20, 21, 48).

For example, a high number of hypomethylated immune genes have a significant role in the development of SLE, as evidenced by the low methylation levels of *IFI44L* at CpG sites in the DNA of SLE patients, Yeung, K.S., et al. performed a genome-wide DNA methylation analysis of patients with systemic lupus erythematosus and found that the IFI44L gene was undermethylated compared to healthy controls (48). Luo, S., et al. found an interesting finding regarding the promoter region of *IFI44L* in naive CD4+ T cells of SLE patients. They observed a lack of methylation at the CG site, which aligns with the SLE Disease Activity Index (SLEDAI) scores. Conversely, it is interesting that *IFI44L* revealed upregulation in CD4+ T cells, indicating that the SLE had no impact on the hypomethylation of naive CD4+ T cells in patients (20). This may be due to the lack of transcription factors required for the *IFI44L* in naive CD4+ T cells. In epigenetics, the existence of *IFI44L* with low methylation induces naive CD4+ T cells to activate each other for a prompt type I IFN response (20). The results indicate that some ISGs such as *IFI44L* may react to IFN-I during T cell activation. This provides evidence for hyper-responsiveness to IFN-I in T cells (20). Similarly, *IFI44L* was found to be hypomethylated at several CpG sites in CD19+ B cells, researchers performed RNA sequencing on whole blood and CD19+ B cells in a separate investigation of primary SS and found that interferon (IFN) -induced genes were significantly hypomethylated (16). It is markedly elevated in mononuclear cells in SLE patients, it can also be regulated by STAT3 in monocytes (21). Based on the ChIP-qPCR assay, it was observed that the binding of the *IFI44L* promoter to STAT3 was increased, while the DNA methylation level of *IFI44L* decreased substantially in STAT3 overexpressed monocytes (21). Therefore, to explore the significance of *IFI44L* upregulation in monocytes associated with SLE, researchers transfected and overexpressed *IFI44L* into monocyte-derived DCs (Mo-DCs) to induce differentiation. The results indicated an increase in the mRNA levels of CD40, CD80, CD83, and CD86. The co-culture of naive CD4+ T cells and Mo-DCs cells led to a remarkable upregulation of IFN-γ and IL-17α This finding suggests that *IFI44L* may be required to maintain the Th1/Th17-related cytokines (21). This study showed that STAT3 can cause DNA demethylation of the *IFI44L* promoter, thereby regulating its expression. Overexpression of *IFI44L* can sustain the upregulation of Th1/Th17-related cytokines (21). Zhao, L., et al. investigated the methylation status of the *IFI44L* gene in SLE patients’ peripheral blood cells using flow cytometry. In resting naive B cells (rNAV) of SLE patients, it was noted that the promoter of *IFI44L* had hypomethylation of two sites (57.4 vs.27.1 and 93.6 vs.82.02), which suggested that the B lymphocytes of SLE patients suffer epigenetic alterations early in their growth process (49).

Moreover, Zhao, L., et al. found that IVRPIE (a kind of lncRNA) could promote the transcription of *IFI44L* by affecting the methylation of its transcription start site (50). Following that, heterogeneous nuclear ribonuclear protein U (hnRNP U) was silenced, and *IFI44L* expression was markedly reduced in IVRPIE overexpressing cells, as shown by RT-qPCR analysis. This finding suggests that hnRNP U is involved in the IVRPIE modulation of *IFI44L* transcripts (50). HBV carriers are early-onset types of liver cancer, and their genomes exhibit high enrichment of genes on immune-related pathways. In the peripheral leukocytes of patients with early-onset HCC, the cg06872964 probe was found to be located in the proximal promoter of *IFI44L* and detected methylation in this region (13). In contrast to the healthy control group, liver cancer patients show a reduced expression of *IFI44L*. This gene not only impedes the metastasis, invasion, and resistance of tumor cells to doxorubicin but also restricts their migration and invasion via the met/Src signaling pathway (51). Collectively, the progression of numerous diseases has been linked to the methylation of *IFI44L*.

### 2.3 Other mechanisms and functions

Multiple studies have demonstrated that *IFI44L* has functions in the formation and modulation of microenvironments. A recent study by Woeckel, V.J., et al. discovered that human osteoblasts reveal sensitivity to the IFN (52). Osteoblasts can stimulate the production of the IFN target gene *IFI44L*. The ECM is an extremely complex environment that includes several chemicals such as fibrin, which play a vital role in regulating cell growth and differentiation (53). In the early stages of human osteoblast differentiation, the formation of ECM is influenced by the immune cytokine IFN-β, leading to a decrease in mineralization. Therefore, *IFI44L* may have a certain therapeutic effect in the early stage of osteoblast differentiation (52). Furthermore, *IFI44L* expression was substantially upregulated in fibroblasts damaged in the heart, indicating that type I INFs may be involved in the damage to heart fibroblasts (54). In a high-throughput sequencing study of *Channel Catfish* (*Ictalurus punctatus*), the researchers found that *IFI44L* might have a significant impact on immune activity and ion transport. However, the precise mechanism underlying this remains unexplored (55).

## 3 Functions of *IFI44L* in human diseases

This gene has significant implications and shows promising potential as both a diagnostic marker and therapeutic target for various human diseases. Next, a detailed analysis was carried out about two areas of malignant tumors and non-neoplastic diseases.

### 3.1 *IFI44L* in malignant tumors

#### 3.1.1 Solid tumors

Researchers identified *IFI44L* as the Hub gene in the protein-protein interaction (PPI) network and a substantially elevated gene in peripheral blood mononuclear cells (PBMCs) by analyzing microarray expression data of all solid tumors (3). When IFI44L is overexpressed, patients’ survival rates are higher, thusthis gene has the potential to function as an immune-related suppressor in tumor therapy (3).

Skin cancer is a very common tumor and its prevention is very crucial. Current scientific research primarily centers around developing effective strategies for its prevention (56, 57). The *CDKN2A* gene mutation is linked to an increased vulnerability to skin cancer. Compared with normal control cells, in *CDKN2A* mutant cell lines, the expression of several immune genes, including *IFI44L*, is significantly increased. This suggests that dysregulation of the transcriptome serves as an important marker for the early progression of skin cancer (58).

Oral cancer (OC) ranks as the 11th most prevalent tumor globally, with approximately 50% of all head and neck tumors originating from the mouth (59, 60). Reyimu, A., et al. observed that *IFI44L* was highly expressed in OC in contrast to normal tissue (2). Based on the results of COX regression analysis, *IFI44L* was identified as an independent prognostic marker of overall survival (OS). Furthermore, receiver operating characteristic (ROC) analysis yielded an area under the curve (AUC) of 0.802 for *IFI44L*, which differentiated OC tissue from normal tissue, indicating that it may serve as a potential predictive biomarker for OC (2).

A tumor known as NPC has been linked to EBV infection (61). Correlation with INF signaling pathways and regulation by miR-9 (34, 62, 63) led to the discovery that *IFI44L* was substantially upregulated in NPC cells and tissues; Thus, it may represent a possible target for modulating the advancement of NPC. Moreover, *IFI44L* was also highly expressed in laryngeal carcinoma HEp2 cells (6). Increased *IFI44L* expression might facilitate apoptosis mediated by IFN α-1a (6).

Early diagnosis of lung cancer (LC) is challenging due to its typically poor prognosis. Some new treatment methods or diagnostic markers are urgently needed to solve this problem (64). Zeng, Y., et al. found a strong association between *IFI44L* and LC tumor immune cells (7). A prognostic model was then developed to assess the prognosis of lung cancer patients using ten *IFI44L*-associated immune modulators. This model yielded an accurate AUC, with all results exceeding 0.75 in a three-year ROC analysis, and was capable of predicting the OS rate for patients with lung adenocarcinoma (LUAD). The prognosis and survival of patients are positively correlated with *IFI44L* expression. Further, cell function experiments showed that high expression of *IFI44L* inhibited the propagation, migration, and penetration of LC cells (7). In addition, *IFI44L* was significantly downexpressed in lung tumorigenesis, and its low expression may be associated with DNA methylation. It was found to facilitate tumor cell apoptosis and may be a tumor suppressor gene through involving the JAK/STAT1 pathway (14). Based on these findings, *IFI44L* is a potent diagnostic marker for LC and may have immunotherapeutic effects.

The HCC is the most prevalent primary malignant tumor (65). Based on the studies, methylation of the proximal promoter of *IFI44L* is strongly associated with the early advancement of LC (13). Huang, W.C., et al. revealed that HCC cells display low levels of *IFI44L* and that low expression is associated with a poorer prognosis for the tumor. Low *IFI44L* expression may facilitate the invasion and metastasis of LC cells via the Met/Src signaling pathway (51). These studies suggest that *IFI44L* is a promising therapeutic target and biomarker.

The prognosis for pancreatic cancer is unfortunately very poor, which makes it the primary cause of cancer-related deaths (66). There is a significant correlation between *IFI44L* overexpression and poorer prognosis among patients with pancreatic ductal adenocarcinoma (PDAC) (p = 0.0153) (12). Furthermore, it may affect pancreatic cancer growth through mechanisms of cell-matrix adhesion and extracellular matrix. These results suggest that *IFI44L* could potentially serve as a new biomarker for PDAC.

Osteosarcoma (OS) is the most prevalent primary malignant bone tumor, characterized by its high aggression and the tendency for metastasis (67). Bone has a highly specific immune environment, and many immune signal molecules play a significant role in bone homeostasis (68). For example, *IFI44L* was found to be decreased in OS and be related to the higher survival rate (p = 0.022) (35). The miR-628-5p, classified as a microRNA, inhibits the expression of *IFI44L*, thereby promoting the propagation, infiltration, and migration of MG-63 cells (35). This result suggests that miR-628-5p may function as a tumor suppressor in OS.

The survival rate of patients with primary central nervous system lymphoma (PCNSL) is another factor associated with *IFI44L* (69). Takashima, Y., et al. developed a prognosis prediction formula that could effectively differentiate between patients with poor and favorable prognoses. The formula including *IFI44L*, showed promising results with an AUC of 0.76 for 2 years and 0.69 for 5-year survival, as evaluated by ROC analysis (69). These results demonstrated that *IFI44L* may hold potential as a therapeutic target for PCNSL.

#### 3.1.2 Hematological malignancy

Acute lymphoblastic leukemia (ALL) is a hematological malignancy that predominantly affects children (70). A prognostic risk model was developed using *IFI44L*, which displays a commendable predictive value ((AUC) for predicted survival at 1, 3, and 5 years is ≥ 0.7 in the datasets examined). In the high-risk group and early relapse patients, *IFI44L* expression was reduced (71). Thus, this model may serve as a novel tool for predicting the prognosis and early detection of ALL recurrence.

Myelodysplastic syndrome (MDS) is a hematologic diease with a high probability of developing into acute leukemia (72). They observed that patients with high *IFI44L* levels in MDS had a more unfavorable prognosis (73), additionally, it may affect pancreatic cancer growth through mechanisms of cell-matrix adhesion and extracellular matrix, suggesting that *IFI44L* could serve as a valuable and significant survival indicator in MDS. It is evident that *IFI44L* exhibits varying expression patterns in different types of cancer. This is because different types of cancer exhibit genomic instability changes at both the early and late stages of tumor development (74). Gene expression is regulated at multiple levels, such as transcription, mRNA stability, mRNA translation, and post-translational modifications (75). Consequently, how these regulations affect protein levels and cancer progression remains a focal point of research in each cancer field. Although gene expression may differ, in certain tumors, integrating other diagnostic methods (such as imaging studies, pathological examinations, and multi-gene panel testing) for a comprehensive assessment can still enhance the accuracy of diagnosis.

In general, *IFI44L* has a significant involvement in malignant tumors. A huge number of genome sequencing studies have revealed that it is associated with the formation of tumors, can alter tumor growth and migration, and is associated with patient prognosis. The expression level of the *IFI44L* gene can be used to predict patient survival rates, and it has the potential to be a valuable diagnostic and prognostic marker.

### 3.2 *IFI44L* in non-neoplastic diseases

Besides the involvement of *IFI44L* in tumors, it is frequently observed in non-tumor conditions, primarily immune disorders, viral infections, and bacterial infectious diseases.

#### 3.2.1 Autoimmune disease

##### 3.2.1.1 Systemic lupus erythematosus (SLE)

It is an autoimmune disease with a very complex pathogenesis, mostly caused by autoimmune defects (76). Multiple studies have demonstrated the significant role of IFN-α in SLE. Among these studies, *IFI44L* has emerged as a key gene with promising potential as a biomarker for diagnosis and treatment (1, 8, 28, 77–79). Zhao, X., et al. found that *IFI44L* had AUC values ≥ 0.8 in ROC analysis when distinguishing SLE from healthy patients (80). Fan, H., et al. validated that *IFI44L* expression in SLE patients trended higher in comparison to the healthy control group (81). More interestingly, *IFI44L* can also be induced by estrogen, suggesting that gender differences should be considered when diagnosing SLE (81). It has been revealed that low methylation genes comprise the majority of the type I INF pathway, with *IFI44L* being particularly significant due to its close association with the development of SLE (48, 82–84). It was found that in the immune cells of SLE patients, including T cells, monocytes (85), granulocytes, or B cells, the CpGs site of *IFI44L* display different degrees of methylation (49, 86, 87). DNA demethylation of the *IFI44L* promoter can be induced by TET2 (which is recruited by STAT3), leading to increased *IFI44L* expression and SLE via stimulation of Mo-DC maturation (21). Different *IFI44L* methylation levels show significant differences between SLE patients and healthy controls or other immune diseases like rheumatoid arthritis (RA). This distinction is highly accurate and reliable, with a sensitivity of 0.81 and 0.84 for SLE and RA patients, respectively (83). The results suggest that *IFI44L* methylation could serve as a valuable epigenetic diagnostic biomarker for SLE (8, 88). Moreover, the methylation of *IFI44L* can also distinguish between discoid lupus erythematosus (DLE) and SLE (89). It was demonstrated by Zhang, B., et al. that *IFI44L* was less methylated than the healthy control and the DLE groups. It can distinguish between SLE from healthy individuals (specificity; 0.98 and sensitivity; 0.96) and SLE from DLE (specificity; 0.7255 and sensitivity; 0.96) when the methylation threshold is set to 25% (89). When the methylation level of *IFI44L* reaches 0.329, its promoter can distinguish SLE from primary antiphospholipid syndrome (APS) (sensitivity; 0.93 and specificity; 0.8) (90). The identification of *IFI44L* has major implications for the diagnosis of SLE and provides significant possibilities for the advancement of targeted therapies in the treatment of SLE patients (91).

##### 3.2.1.2 Sjogren’s Syndrome (SS)

Sjogren’s Syndrome (SS) is an autoimmune disease with an unclear pathogenesis (92). Studies have shown that SS is also associated with some IFN genes such as *IFI44L* (93–95), the hyperactivation of the IFN system is recognized as a crucial factor in the development of disease (96). Similar to SLE, *IFI44L* is also found to be hypomethylated in naive CD4+ T cells of SS patients, indicating its involvement in the development of SS (4). Strong associations between *IFI44L*, a component gene of the IFN type I signature, and myxovirus-resistance protein A (MxA), which can evaluate the activity of SS, suggest that *IFI44L* may also be capable of measuring the activity of SS (97). Chen, S., et al. found that low methylation of IFN-associated genes such as *IFI44L* is a common feature of T cells and monocytes in many immune systems diseases such as SLE, RA, and systemic sclerosis (SSc), and may function as biomarkers to differentiate and diagnose these diseases (98, 99). Significant hypomethylation of *IFI44L* was also observed in whole blood and CD19+ B cells of SS patients (16). Salivary samples and salivary gland biopsy samples from patients diagnosed with SS showed significant differences in *IFI44L* expression. These findings indicate that *IFI44L* is present not only in peripheral blood but also in saliva and salivary glands. Thus, *IFI44L* has the potential to serve as a potent diagnostic biomarker for SS (100).

##### 3.2.1.3 Rheumatoid *arthritis* (RA)

DNA methylation sites that are connected with RA are present in ISGs (101). Cooles, F.A.H., et al. found that the IFN gene signature (IGS) may influence the epigenetic regulation of lymphocytes in RA patients (19). In B cells and T cells, the levels of IGS and IFN-α are correlated with varying degrees of methylation site abundance; IGS serves as an indicator of IFN-α protein expression. When the IGS score is increased, the prognosis of patients is worse, suggesting that IGS may be a good prognostic biomarker in RA (19). Thousands of molecules were screened by Yadalam, P.K., et al. to determine that *IFI44L* is a drug target for RA. Vemurafenib is the most suitable drug for the *IFI44L* target, and the combination of the two has good stability. Vemurafenib may be an anti-inflammatory drug for treating this disease (18). In RA patients, *IFI44L* primarily regulates the IFN signaling pathway via DCs, resulting in a crucial molecular target (42). Moreover, the IFN signature (including *IFI44L* and other genes) can predict the sensitivity of RA patients to rituximab (RTX) with excellent ROC analysis (an AUC of 0.87) (102).

##### 3.2.1.4 Other autoimmune diseases

In addition to its primary association with SLE, RA, and SS, *IFI44L* also has a significant impact on various other autoimmune diseases. Different levels of methylation were also found at the CpGs site of *IFI44L* in monocytes and macrophages of patients with SSc, suggesting that *IFI44L* contributes to the development of this disease (103–105). Considerable expression of *IFI44L* was observed in lung microvascular endothelial cells (MVECs) during the study of SSc-induced vascular lesions, this result may indicate the role of *IFI44L* in the vasculopathy caused by SSc (106). However, very few studies have investigated the molecular mechanism of mixed connective tissue disease (MCTD) and the researchers found the epigenetic signal of *IFI44L* and confirmed that it is related to the heritability of MCTD. The diagnostic AUC value reached 0.97, 0.9, and 0.84 when distinguishing this disease from healthy people, RA, and SSc patients, suggesting the potential of *IFI44L* as an epigenetic biomarker for the MCTD (107). Lerkvaleekul, B., et al. found that *IFI44L* was also associated with clinical activity in juvenile dermatomyositis (JDM) (108). The *IFI44L* gene has substantial upregulation in CD4+ and CD8+ T cells of patients with large-vessel involvement in giant cell arteritis (LV-GCA). This upregulation may potentially be linked to the pathogenesis of the disease (109). The *IFI44L* gene is differentially expressed in both membranous nephropathy (MN) and lupus nephritis (LN). It is a member of a group of differentially expressed genes (DEGs) that has a high diagnostic value in differentiating patients with MN from those with LN (110).

#### 3.2.2 Bacterial and viral infectious diseases

The *IFI44L* gene can participate in virus and bacterial replication (39, 111). It is frequently observed as a DEG in various diseases caused by bacteria and viruses. Its presence is significant in the progression of these diseases (17, 112). It can also distinguish some diseases infected by bacteria and viruses (113), such as acute febrile illness caused by viruses or bacterial infections (114), and thus, *IFI44L* can significantly contribute to the differential treatment of febrile diseases.

To differentiate between bacterial and viral infections, Herberg, J.A., et al. identified the *IFI44L* gene for the first time in 2016 (115). In a Disease Risk Score (DRS) composed of *IFI44L* and *FAM89A*, *IFI44L* was highly expressed in patients with bacterial infections. The AUC of this DRS exceeded 0.89 when distinguishing bacterial infections, viral infections, and inflammatory diseases (115). Following this, Pennisi, I., et al. used a disposable diagnostic cartridge for clinical detection using semiconductor sensing technology and the DRS; this represents a significant breakthrough and invention in the detection of viral and bacterial infections (116). Moreover, they observed that *IFI44L* could differentiate between bacterial and viral infections with an AUC of 0.97. Further, *IFI44L* expression was reduced during the acute infection phase compared to the recovery phase, indicating that the dynamic change of *IFI44L* could serve as a prognostic indicator for the therapeutic efficacy of the disease (114). Experimental studies revealed that when *IFI44L* was knocked out, the survival rate of cells infected with *Mycobacterium Tuberculosis* increased. Similarly, when the gene was highly expressed, the survival rate of cells increased; these results suggest that *IFI44L* has effective antibacterial activity and may clear the burden of Mycobacterium tuberculosis from macrophages (27).

Overexpression of *IFI44L* can affect the activity of many viruses. For instance, when lung epithelial cells are infected with respiratory syncytial virus (RSV), the mRNA level of *IFI44L* is substantially upregulated. This upregulation in *IFI44L* can effectively hinder the replication of the virus and lower the stimulation of the IFN pathway caused by respiratory viruses. These results suggest possible possibilities for therapeutic interventions in RSV (117, 118). In germ cells, overexpression of *IFI44L* can inhibit the production of Zika virus (ZIKV) (119). The COVID-19 pandemic, caused by severe acute respiratory syndrome coronavirus 2 (SARS-CoV-2), has had a significant impact globally (120). In PBMCs infected by SARS-CoV-2, *IFI44L* is upregulated and involved in some immune responses (121–123). Patients infected with COVID-19 showed varying levels of methylation in *IFI44L* (124). Expression of *IFI44L* from endothelial cells was also increased in SARS-CoV-2-infected heart tissue, and this upregulation was found to be related to the INF pathway (125). These outcomes indicate that *IFI44L* could be a promising target for treating SARS-CoV-2 infections. Moreover, differential upregulation of *IFI44L* has been found in cells or tissues infected by many other viruses such as Hepatitis E virus (HEV), Influenza A virus (IAV), and rhinoviruses (RV), indicating that *IFI44L* holds significant promise for treating diseases caused by viral infections (126–128). Another interesting finding of Brochado et al. revealed that the *IFI44L* showed a pattern of overexpression in most antiviral responses; however, it was one of the most downregulated genes in patients co-infected with HIV and HCV (17, 129). Similarly, they also found that *IFI44L* expression was downregulated in HIV-infected macrophages, possibly due to HIV’s strong resistance to IFN-mediated immune responses (130).

#### 3.2.3 Other systemic diseases

In addition to the above-mentioned diseases, *IFI44L* also has a significant impact on various other system diseases, such as those affecting the nervous, cardiovascular, and endocrine systems. Many inflammation-related genes, including *IFI44L*, are downregulated in the brain tissue of patients with Major Depressive Disorder (MDD), underscoring the role of neuroinflammation in MDD (131). Significant associations were also observed between a risk score model (including *IFI44L*) and the clinical symptoms of patients diagnosed with bipolar disorder and schizophrenia (132). Feenstra et al. identified *IFI44L* on chromosome 1p31.1 as the initial locus for febrile seizures related to the MMR vaccine. They recognized *IFI44L* as a gene linked to febrile seizures unrelated to the MMR vaccine (p = 1.2 × 10^-9^ versus controls and p = 5.9 × 10^-12^ versus controls, respectively) (133). In diabetes and diabetes-related complications such as diabetic foot ulcer (DFU) and proliferative diabetic retinopathy (FDR), *IFI44L* has been listed as an immune-related marker and a possible therapeutic target for metformin (134–136). Analysis of the whole genome of leukocytes in obese patients revealed an association between IFI44L and body mass index (BMI). This suggests that IFI44L may serve as a potential biomarker in the diagnosis of obesity (137). The *IFI44L* was found to be strongly associated with cardiovascular disease. Its expressions were found to be greatly increased and strongly associated with myocardial infarction (MI) in the heart tissues of rat MI models. The expression of *IFI44L* was also effectively suppressed by metoprolol, indicating that it could serve as a potential marker for human ischemic cardiomyopathy (ISCM) (138). It can mediate fibroblast damage of the fetal heart in SSA/Ro autoantibody-associated congenital heart block (CHB) (54), indicating that *IFI44L* can serve as a potential clinical intervention target. When circulating endothelial progenitor cells (EPCs) were exposed to monomeric C-reactive protein (mCRP), there was a significant upregulation of interferon-responsive genes, including *IFI44L* (139). It is also differentially expressed in ischemic stroke and pulmonary arterial hypertension (PAH), revealing its potential as a therapeutic target in PAH (140, 141). It is highly expressed or functions as a hub gene in the PPI network (5, 142–146), in several chronic inflammatory diseases, including airway inflammation, periodontitis, neuritis, psoriasis, necrosis of the femoral head, and parasitic diarrhea. This gene association may have implications for innate immune response pathways and inflammation. It is also involved in the pathogenesis of premature ovarian failure (POF) as a necrotic gene (147).

## 4 Discussion and perspective

When bacteria, viruses, and tumor cells infiltrate the body, IFN can trigger the activation of multiple ISGs within the host cell. These ISGs then produce proteins that serve specific biological functions, and these proteins can independently or synergistically participate in a wide range of vital processes (148, 149). They are very important immune-related regulatory genes (150). The functions of these ISG products are highly varied to impeding viral replication, growth and migration of tumor cells, stimulating the propagation of immune cells, enhancing cytotoxicity via regulation of multiple signaling pathways, facilitating protein synthesis and modification (151–155). Among them, the antiviral effect stands out as the most crucial and extensively studied. However, limited research has been conducted on tumors and autoimmune diseases. Recently, an increasing number of studies have revealed the significant impact of ISG in the field of tumor and immune function.

As an ISG, *IFI44L* has antiviral, antibacterial, and antitumor properties comparable to those of other ISGs. This review also provided a summary of the other crucial functions of *IFI44L*, including its involvement in ECM formation, conduction of cardiac electrical signals, and induction of cell apoptosis, along with signal transduction and methylation modification (6, 52, 54, 55). Unfortunately, many investigators have only discovered that *IFI44L* influences the growth, migration, and apoptosis of tumor cells. The precise mechanism by which it acts in the tumor immune environment has not been fully investigated. The clinical development of anti-tumor drugs has resulted in a degree of resistance. Several genes have been extensively researched as potential targets for drug development. However, *IFI44L* stands out due to its ability to evaluate a patient’s response to anti-tumor drugs like doxorubicin (156). It is a key gene in SLE, SS and RA. When it relates to exploring autoimmune diseases, the *IFI44L* has proven to be incredibly valuable. It has a crucial role in diagnosing diseases, differentiating patients, and assessing prognosis. As a result, it has become a promising candidate for both diagnostic markers and therapeutic targets in various diseases (30). However, in many studies, *IFI44L* is only one of the genes that constitute a prognostic risk model. It evaluates disease prognosis along with other immune-related genes, which is not very accurate in assessing the effect of *IFI44L* independently. Furthermore, *IFI44L* manifests a favorable impact on resistance to bacteria and viruses. While HIV acquires a certain degree of resistance to it, it is still capable of inhibiting the replication of other viruses. Further study is needed to better understand its inhibitory effect on HIV (130). Moreover, *IFI44L* has been linked to certain neurological, cardiovascular, chronic inflammatory, and endocrine diseases (131, 134, 138, 142).

In the application of clinical diagnosis, *IFI44L* can be used as a potential target for tumor drug development, as well as a differential target for bacterial and viral diagnosis. In addition, *IFI44L* can also be used as a tumor prognostic model to estimate the survival rate of patients, playing an important role in disease diagnosis and treatment. However, the molecular mechanisms underlying *IFI44L*’s role in disease development have not been extensively studied. Most current research focuses on the surface-level relationship between expression levels and disease progression, failing to fully uncover its intrinsic biological processes and regulatory networks. Although a few studies have explored the regulatory pathways of *IFI44L* and provided some valuable insights, these findings have yet to be effectively translated into clinically practical diagnostic markers, limiting their potential application in medical practice. Additionally, the significant specificity of *IFI44L* expression in various types of tumors and immune diseases makes it a promising tool for distinguishing patients from healthy individuals. However, due to the lack of tissue specificity, accurate clinical diagnosis often requires the combination of other auxiliary diagnostic methods. Furthermore, there are limited reports in the existing literature on the sensitivity value of *IFI44L* in early diagnosis, suggesting that future research should pay more attention to its sensitivity performance at different stages of disease. This would better assess its potential as an early diagnostic marker. Through further research and technological advancements, *IFI44L* has the potential to be developed into an efficient and reliable clinical diagnostic tool, providing robust support for the early detection and treatment of diseases.

## 5 Conclusion

In summary, *IFI44L* emerged as a promising biomarker in studies examining a range of diseases. A limited number of studies investigated the relationship between *IFI44L* and diseases. Its application in clinical practice was also uncommon. Few studies developed *IFI44L* into a disposable diagnostic cartridge for use in clinical settings. Despite this illustrating that *IFI44L* has the potential to yield results for clinical applications, the current pool of research is quite limited. In the future, more research is required regarding the therapeutic and diagnostic potential of *IFI44L*, not only for its application in drug development but also in clinical diagnosis.

## Glossary

IFI44L: Interferon-induced protein 44-like
ISGs: interferon-stimulated genes
OC: oral cancer
RCC: renal cell carcinoma
SS: Sjogren’s syndrome
SLE: systemic lupus erythematosus
PDAC: pancreatic ductal adenocarcinoma
HCC: hepatocellular carcinoma
LC: lung cancer
ECM: extracellular matrix
pDCs: plasmacytoid dendritic cells
TBK1: TANK-binding kinase
LMKB: Limkain B
HUVECs: human umbilical vein endothelial cells
3′-UTR: 3’ untranslated region
NPC: nasopharyngeal carcinoma
OS: osteosarcoma
RIPK3: serine/threonine-protein kinase 3
JEV: Japanese encephalitis virus
IFNLP: IFN-like protein
IFN: Interferon
DCs: dendritic cells
HIV-1: human immunodeficiency virus type 1
siRNA: short interfering RNA
HBV: hepatitis B virus
cSLE: Childhood-onset SLE
GMECs: goat mammary epithelial cells
SLEDAI: SLE Disease Activity Index
Mo-DCs: monocyte-derived DCs
rNAV: resting naive B cells
hnRNP U: heterogeneous nuclear ribonuclear protein U
PPI: protein-protein interaction
PBMCs: peripheral blood mononuclear cells
OC: Oral cancer
OS: overall survival
ROC: receiver operating characteristic
AUC: area under the curve
LC: lung cancer
LUAD: lung adenocarcinoma
PCNSL: primary central nervous system lymphoma
ALL: Acute lymphoblastic leukemia
MDS: Myelodysplastic syndrome
RA: rheumatoid arthritis
DLE: discoid lupus erythematosus
APS: antiphospholipid syndrome
MxA: myxovirus-resistance protein A
SSc: systemic sclerosis
IGS: IFN gene signature
RTX: rituximab
MVECs: microvascular endothelial cells
MCTD: mixed connective tissue disease
LV-GCA: large-vessel involvement in giant cell arteritis
MN: membranous nephropathy
DEGs: differentially expressed genes
DRS: Disease Risk Score
RSV: respiratory syncytial virus
ZIKV: Zika virus
SARS-CoV-2: severe acute respiratory syndrome coronavirus 2
HEV: Hepatitis E virus
IAV: Influenza A virus
RV: rhinoviruses
MDD: Major Depressive Disorder
DFU: diabetic foot ulcer
FDR: proliferative diabetic retinopathy
BMI: body mass index
MI: myocardial infarction
ISCM: ischemic cardiomyopathy
CHB: congenital heart block
EPCs: endothelial progenitor cells
mCRP: C-reactive protein
PAH: pulmonary arterial hypertension
POF: premature ovarian failure
